# Epigallocatechin gallate (EGCG) attenuates severe acute respiratory coronavirus disease 2 (SARS-CoV-2) infection by blocking the interaction of SARS-CoV-2 spike protein receptor-binding domain to human angiotensin-converting enzyme 2

**DOI:** 10.1371/journal.pone.0271112

**Published:** 2022-07-13

**Authors:** Tomokazu Ohishi, Takayuki Hishiki, Mirza S. Baig, Sajjan Rajpoot, Uzma Saqib, Tomohiko Takasaki, Yukihiko Hara

**Affiliations:** 1 Institute of Microbial Chemistry (BIKAKEN), Numazu, Microbial Chemistry Research Foundation, Numazu-shi, Shizuoka, Japan; 2 Kanagawa Prefectural Institute of Public Health, Chigasaki, Kanagawa, Japan; 3 Department of Biosciences and Biomedical Engineering (BSBE), Indian Institute of Technology (IIT), Simrol, Indore, India; 4 Department of Chemistry, Indian Institute of Technology (IIT), Simrol, Indore, India; 5 Tea Solutions, Hara Office Inc., Sumida-ku, Tokyo, Japan; Cornell University, UNITED STATES

## Abstract

The outbreak of the coronavirus disease 2019 caused by the severe acute respiratory syndrome coronavirus 2 triggered a global pandemic where control is needed through therapeutic and preventive interventions. This study aims to identify natural compounds that could affect the fusion between the viral membrane (receptor-binding domain of the severe acute respiratory syndrome coronavirus 2 spike protein) and the human cell receptor angiotensin-converting enzyme 2. Accordingly, we performed the enzyme-linked immunosorbent assay-based screening of 10 phytochemicals that already showed numerous positive effects on human health in several epidemiological studies and clinical trials. Among these phytochemicals, epigallocatechin gallate, a polyphenol and a major component of green tea, could effectively inhibit the interaction between the receptor-binding domain of the severe acute respiratory syndrome coronavirus 2 spike protein and the human cell receptor angiotensin-converting enzyme 2. Alternately, *in silico* molecular docking studies of epigallocatechin gallate and angiotensin-converting enzyme 2 indicated a binding score of −7.8 kcal/mol and identified a hydrogen bond between R393 and angiotensin-converting enzyme 2, which is considered as a key interacting residue involved in binding with the severe acute respiratory syndrome coronavirus 2 spike protein receptor-binding domain, suggesting the possible blocking of interaction between receptor-binding domain and angiotensin-converting enzyme 2. Furthermore, epigallocatechin gallate could attenuate severe acute respiratory syndrome coronavirus 2 infection and replication in Caco-2 cells. These results shed insight into identification and validation of severe acute respiratory syndrome coronavirus 2 entry inhibitors.

## Introduction

The coronavirus disease 2019 (COVID-19) outbreak emerged as an unexpected crisis, infecting millions of people and claiming lives globally (https://coronavirus.jhu.edu/map.html). COVID-19 was first detected in Wuhan city, Hubei province, China, in December 2019, as a severe pneumonia of unknown etiology [1, 2]. The initial spread of infection occurred at the Huanan wholesale market, potentially due to animal contact, and then spread via human–human viral transmission, reaching epidemic and then pandemic proportions [3, 4]. Severe acute respiratory syndrome coronavirus 2 (SARS-CoV-2) is a strain of coronavirus causing COVID-19 [5]. Many mutations have been reported, and some were identified as variants of concern, such as alpha, beta, gamma, delta, and omicron [6]. Regarding the suppression of COVID-19 infection, several strategies, including antiviral and immune-based therapies, as well as vaccination development have been followed. In this context, many clinical trials have been performed as therapeutic options such as vaccines, potent repurposed drugs, and neutralizing antibodies [7]. Till present, remdesivir, which is a broad-spectrum antiviral nucleotide analog prodrug that inhibits SARS-CoV-2, is the only FDA-approved drug intended to treat COVID-19 [8]. More accurate data and clinical studies supported with convincing strategies are needed to test the effects of other potent agents in the treatment of COVID-19 infection.

The entrance of coronavirus into target cells is facilitated via the viral spike protein; in SARS-CoV-2, the spike glycoprotein, which contains a defined receptor-binding domain (RBD) required for interaction with the host cell receptor angiotensin-converting enzyme 2 (ACE2), regulates both cross-species and human–human infections of SARS-CoV-2 [9]. This interaction between SARS-CoV-2 spike RBD and ACE2 is considered as the critical functional component that facilitates the fusion of the viral membrane with human cells [10–12]. Therefore, disrupting this interaction might demonstrate the potential to inhibit SARS-CoV-2 from entering human cells.

Phytochemicals are natural compounds often found in beverages, vegetables, and fruits such as tea, coffee, wine, turmeric, onions, broccoli, apples, berries, citrus fruits, and plums [13]. Several previous studies have shown that the phytochemical components found in tea, coffee, wine, and curry might demonstrate favorable effects against various diseases comprising cancer, obesity, neurodegenerative disorders, and diabetes [14–17]. In addition, recent molecular docking studies have identified several phytochemicals as potential anti-COVID-19 agents [18]. In this study, we evaluated the inhibitory effects of 10 well-known phytochemicals on SARS-CoV-2 spike RBD binding to ACE2 using enzyme-linked immunosorbent assays (ELISAs); the phytochemicals were as follows: epigallocatechin gallate (EGCG) [19], epigallocatechin (EGC) [19], epicatechin gallate (ECG) [19], epicatechin (EC) [19], chlorogenic acid [20], genistein [21], quercetin [22], curcumin [23], resveratrol [19], and sulforaphane [24]. As a result, EGCG showed the most potential inhibitory effect of spike RBD binding to ACE2, and hence, attenuated the infection of SARS-CoV-2 in Caco-2 cells. These results indicate that EGCG might be a lead natural compound against SARS-CoV-2 for further optimization and development.

## Materials and methods

### Cell lines

We obtained 293FT and Caco-2 cells from the American Type Culture Collection (Manassas, VA, USA) and cultured both cell lines in Dulbecco’s modified Eagle’s medium (Nacalai Tesque, Inc.) supplemented with 10% heat-inactivated fetal bovine serum (Thermo Fisher Scientific Inc., Waltham, MA, USA), 100 units/mL of penicillin, 100 μg/mL of streptomycin, and 0.25 μg/mL of amphotericin B (Nacalai Tesque, Inc.) at 37°C in a humidified atmosphere containing 5% CO_2_.

### Reagents

We purchased EGCG and EC (> 95% purity) from Mitsui Norin Co. Ltd. (Shizuoka, Japan), EGC (11809), ECG (11808), genistein (10005167), and sulforaphane (10496) from Cayman Chemical (Ann Arbor, MI, USA), chlorogenic acid (C9244) and resveratrol (R1776) from LKT Laboratories, Inc. (St. Paul, MN, USA), quercetin from ChemScene (Monmouth Junction, NJ, USA), and curcumin from Enzo Life Science (Farmingdale, NY, USA).

### Enzyme-linked immunosorbent assay (ELISA)

We examined the inhibitory effects of SARS-CoV-2 RBD and ACE2 binding by ELISAs using the Spike S1 RBD (SARS-CoV-2): ACE2 Inhibitor Screening Assay Kit (BPS Bioscience, #79931, San Diego, CA, USA) following the manufacturer’s instructions; we tested all compounds at 100 or 20 μM, and we briefly incubated SARS-CoV-2 spike RBD-Fc (50 ng) overnight for 12 h into a nickel-coated 96-well plate. Afterwards, we washed the plate three times, then incubated for 10 min with a blocking buffer supplied in the kit, then added 10 μL of inhibitor solution containing the selected compound, and incubated for 5 min at room temperature with slow shaking. We used 10 μL of inhibitor buffer (5% dimethyl sulfoxide [DMSO] solution) as a control. Subsequently, we added to each well, except for the blank, His-tagged ACE2 (50 ng) supplied in the kit, and incubated for 1 h at room temperature, with slow shaking. Next, we washed the wells three times and incubated with the blocking buffer for 10 min. Then, we added Anti-mouse-Fc-HRP, supplied in the kit, and incubated for 1 h at room temperature, with slow shaking. Finally, we added a horseradish peroxidase (HRP) substrate supplied in the kit to the wells in order to produce chemiluminescence, which we measured via an EnSpire microplate reader (PerkinElmer, Waltham, MA, USA).

### Docking study

The binding of RBD of the SARS-CoV-2 spike and peptidase domain of ACE2 provided a platform to assess the interactions of their interface residues within a 5-Å region [10, 25, 26]. In this context, we obtained the crystal structure of this interaction from the protein databank file for the RBD/ACE2 complex (PDB: 6M17) and analyzed it using the UCSF Chimera tool [27]. Accordingly, we prepared the structures of ACE2 and EGCG for docking using the AutoDock v4.2 tool. Also, we prepared the ACE2 chain (PDB ID 6M17) by deleting the water molecules and any co-crystallized ligands followed by the addition of polar hydrogen atoms. Finally, we assigned the Kollman charges to the structure and spread them equally to all the atoms before saving the structure in the AutoDock file format pdbqt. Next, we prepared the grid box by covering the entire ACE2 structure. We prepared the ligand EGCG automatically by the AutoDock tool, which added Gasteiger charges and merged the polar hydrogen atoms. Finally, we performed *in silico* docking of EGCG to determine its ability to target ACE2 using the AutoDock Vina tool in unbiased blind docking mode [28]. The three-dimensional and two-dimensional interaction diagrams of EGCG with ACE2 indicate the binding pattern, and thus, we identified the key interacting residues using PyMOL and BIOVIA Discovery Studio Visualizer.

### Western blot analyses

We performed western blot analyses, as described previously [29]. In this context, we briefly lysed the cells in lysis buffer (20 mM HEPES, pH 7.5, 150 mM NaCl, 1% (v/v) Triton X-100, 10% (v/v) glycerol, 1 mM EDTA, 50 mM NaF, 50 mM β-glycerophosphate, 1 mM Na_3_VO_4_, and 25 μg/mL each of antipain, leupeptin, and pepstatin) for 30 min on ice. After centrifugation at 20,400 *g* for 10 min at 4°C, we collected the lysates, separated them by sodium dodecyl sulfate-polyacrylamide gel electrophoresis, transferred them onto polyvinylidene difluoride membranes (Merck KGaA, Darmstadt, Germany), and analyzed them by Western blotting. We measured protein levels using the following primary antibodies: anti-ACE2 antibody (ab273433, 1:1000; Abcam, Cambridge, UK) and anti-β-tubulin antibody (#5346, 1:1000; Cell Signaling Technology, Danvers, MA, USA).

### Proliferation assays *in vitro*

We measured cell proliferation using the CellTiter-Glo Luminescent Cell Viability Assay Kit (Promega, Madison, WI, USA) following the manufacturer’s instructions; we plated the cells (2,000 cells/100 μL/well) in triplicate in 96-well plates and treated them with the indicated concentrations of EGCG. We measured cell viability after 2 h. Also, we measured luminescence using an EnSpire microplate reader (PerkinElmer, Waltham, MA, USA).

### SARS-CoV-2 virus infection assays

We cultured Caco-2 cells in a 96-well plate and infected them with SARS-CoV-2 at a multiplicity of infection (MOI) of 0.5 or 1 in the presence of EGCG at the indicated concentrations. After two to 24 h post-infection (hpi), the cell culture supernatant and/or cell lysate were collected, viral RNA was isolated, and gene expression was measured using quantitative real-time reverse transcription-polymerase chain reaction (RT-qPCR) analysis. For the analysis of 24 hpi, we rinsed the cells three times with phosphate-buffered saline (PBS) and added fresh medium before or after SARS-CoV-2 infection. We performed RT-qPCR analysis of the nucleocapsid region of the SARS-CoV-2 genome using TaqMan Fast Virus 1-Step Master Mix (Thermo Fisher Scientific) and 7500 Real-Time PCR System (Thermo Fisher Scientific). We used the SARS-CoV-2-specific primer-probe sets (forward: AAATTTTGGGGACCAGGAAC, reverse: TGGCAGCTGTGTAGGTCAAC) and TaqMan probe (FAM-ATGTCGCGCATTGGCATGGA-TAMRA) [30]. Next, we plotted standard curves for the SARS-CoV-2-specific genome showing the cycle threshold value vs. log of initial copy number, and we calculated the slope of the standard curve to describe the efficiency of PCR. Additionally, we expressed the obtained values as a percentage of control.

### Statistical analyses

All data are representative of at least three independent experiments. Statistical analysis was carried out using the two-tailed Student’s t-test. All data are presented as the mean ± standard deviation (SD). A p-value < 0.05 is considered statistically significant.

## Results

### EGCG inhibits binding between SARS-CoV-2 spike RBD and ACE2

**Fig 1:**
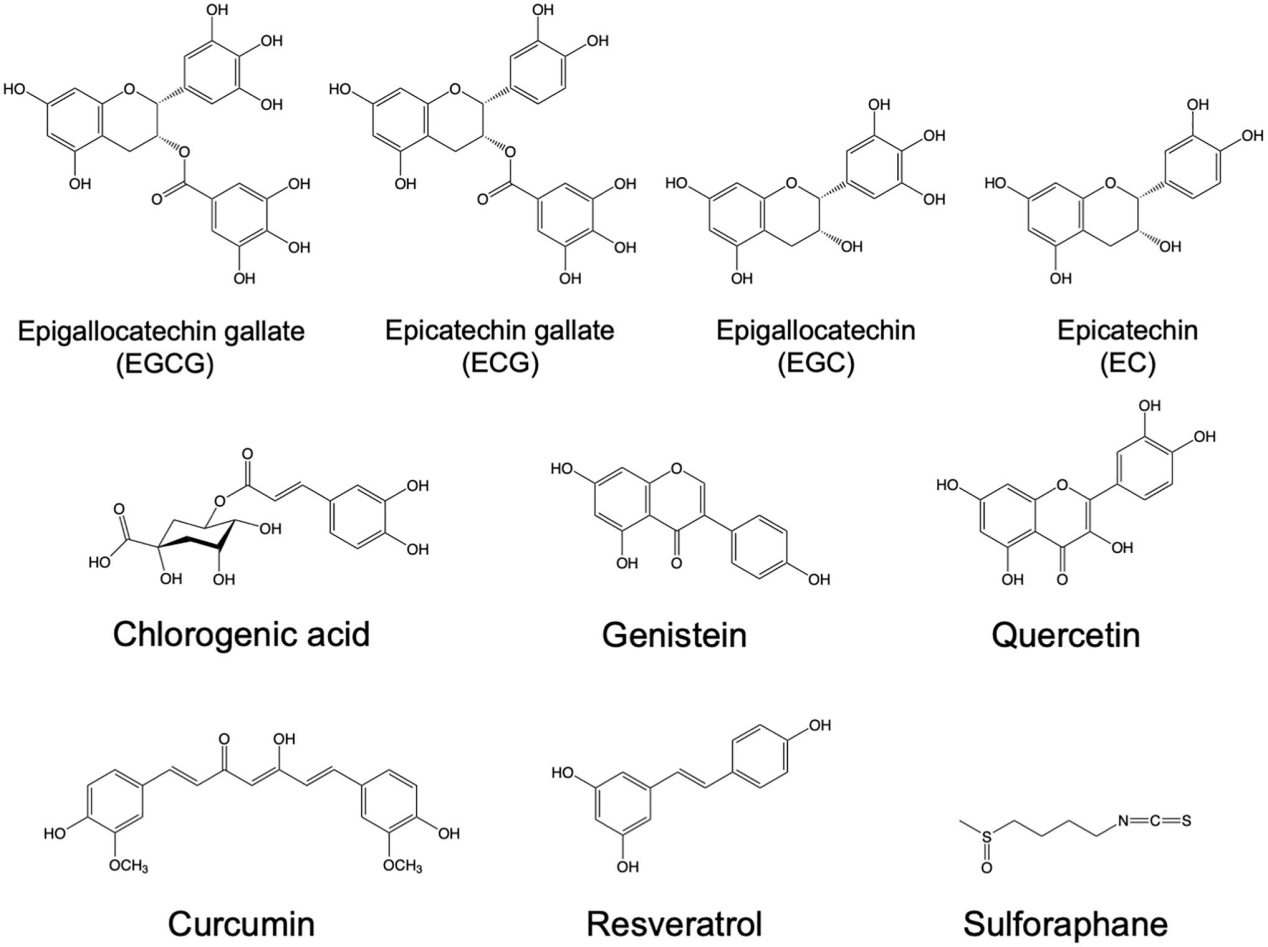
Chemical structures of phytochemicals. 1: Epigallocatechin gallate (EGCG); 2: epigallocatechin (EGC); 3: epicatechin gallate (ECG); 4: epicatechin (EC); 5: chlorogenic acid; 6: genistein; 7: quercetin; 8: curcumin; 9: resveratrol; 10: sulforaphane.

**Fig 2:**
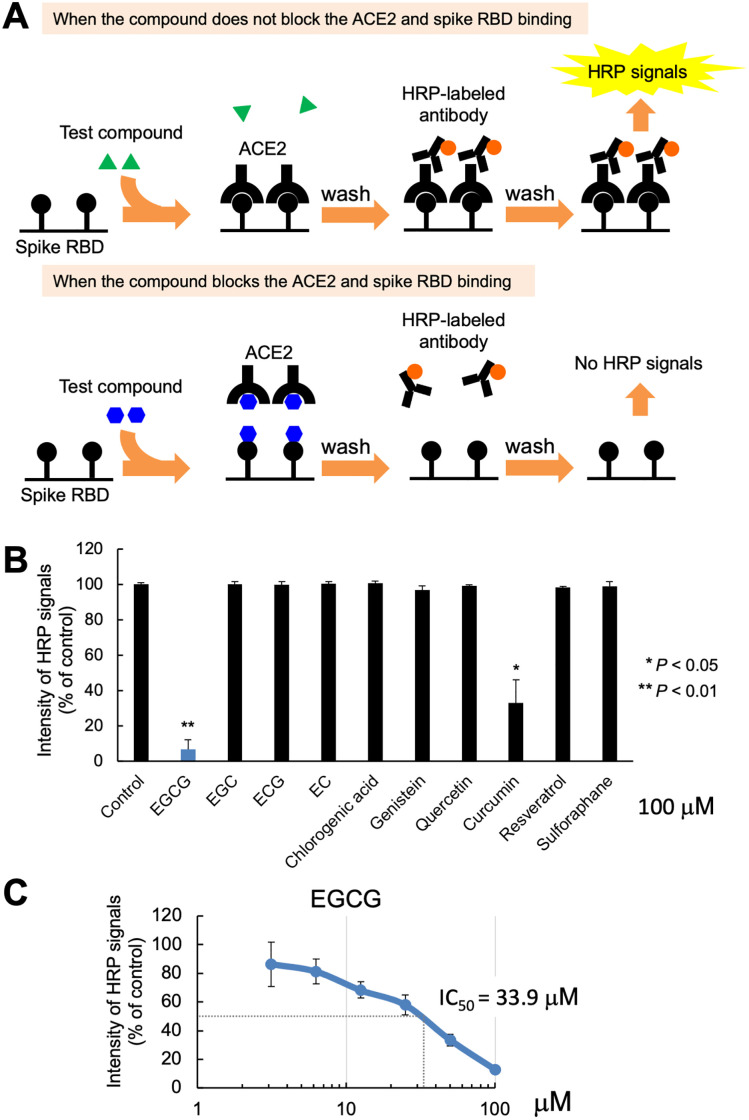
Epigallocatechin gallate (EGCG) inhibits the interaction between the spike receptor-binding domain (RBD) and human cell receptor angiotensin-converting enzyme 2 (ACE2). (A) Schematic of the ELISA-based screening of phytochemical inhibitory activity on ACE2 and SARS-CoV-2 spike RBD binding. Upper scheme: When the compound does not block the ACE2 and spike RBD binding. Lower scheme: When the compound blocks the ACE2 and spike RBD binding. (B) ELISA results of phytochemical inhibition of the ACE2 and spike RBD binding. Low-intensity HRP signals indicate that the compound successfully blocked the ACE2 and spike RBD binding. Values are presented as the mean ± SD. Asterisks indicate the significant difference compared with the DMSO-treated control (**p < 0.01, *p < 0.05). (C) ELISA results of serially diluted EGCG (3.125–100 μM) inhibition of ACE2 and spike RBD binding b. IC50 was calculated using the IC50 calculator (https://www.aatbio.com/tools/ic50-calculator).

Disrupting the SARS-CoV-2 spike binding to ACE2 could inhibit the coronavirus from entering ACE2-expressing human cells [31]. Based on this assumption, we used the ELISA technique to screen the interaction between SARS-CoV-2 spike RBD and ACE2 using a range of the following 10 phytochemicals: EGCG, EGC, ECG, EC, chlorogenic acid, genistein, quercetin, curcumin, resveratrol, and sulforaphane. In this context, we identified the potency of their inhibitory activity (Fig 1); within this framework, 20 μM and 100 μM of EGCG, as well as 100 μM of curcumin, significantly inhibited the binding of spike RBD to ACE2 (*p* < 0.01 and *p* < 0.05, respectively), whereas the other phytochemicals exhibited no significant effect on this interaction. Nevertheless, EGCG demonstrated the highest inhibitory activity against SARS-CoV-2 spike RBD binding to ACE2 (93.3%, 100 μM; 36.6%, 20 μM), whereas curcumin showed moderate activity (67.0%, 100 μM) (Fig 2A and 2B and S1 Fig). Conversely, the half-maximum inhibitory concentration (IC50) of EGCG for binding the spike RBD to ACE2 was 33.9 μM, as shown on the graph plot expressing the inhibition percentage against the concentration of the inhibitor (Fig 2C). Therefore, EGCG might be considered for further investigation as a new modality for the therapeutic intervention against SARS-CoV-2 infection.

### Binding of EGCG with ACE2 might disrupt the SARS-CoV-2 RBD interaction

**Fig 3:**
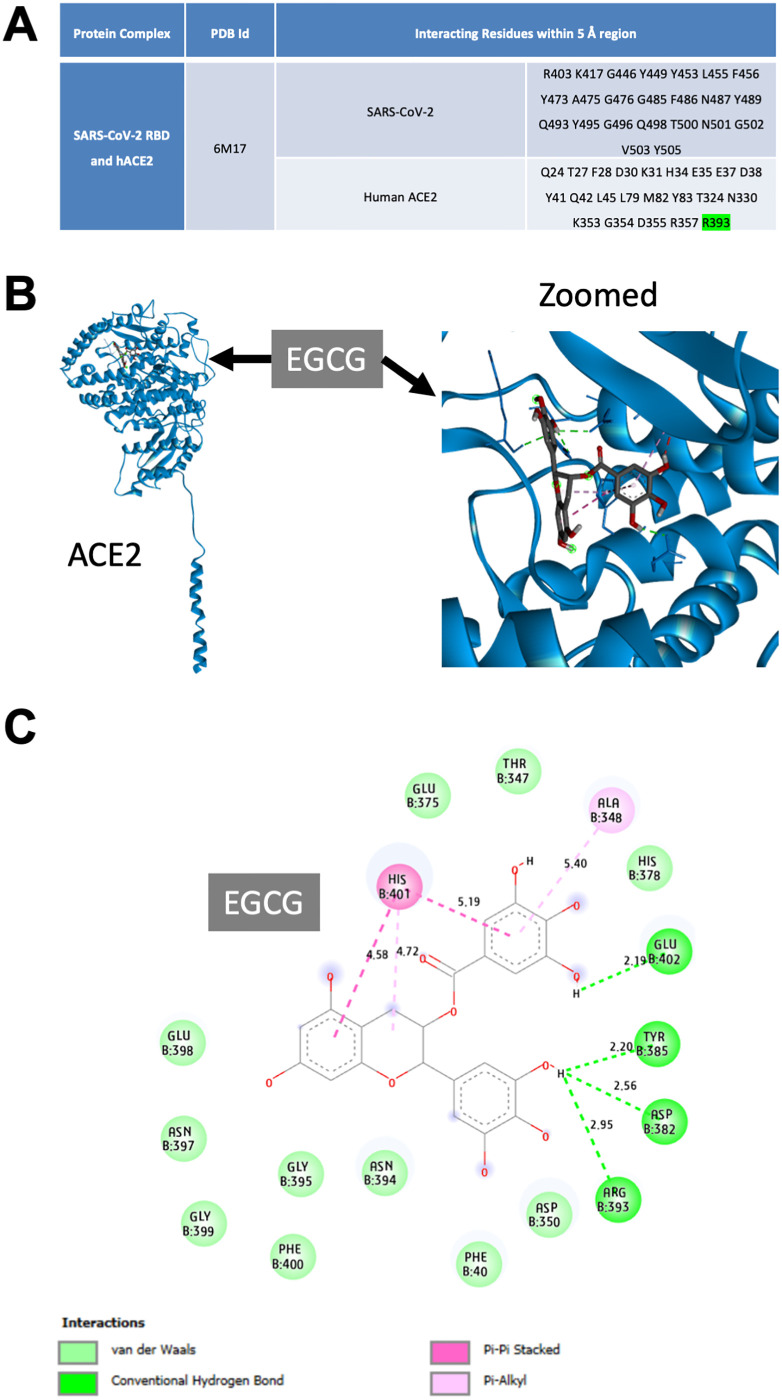
Docking interaction of epigallocatechin gallate (EGCG) with human angiotensin-converting enzyme 2 (ACE2). (**A**) Interacting residues determined within a 5-Å region at the interface of the severe acute respiratory syndrome coronavirus 2 (SARS-CoV-2) receptor-binding domain (RBD) and human ACE2 from their crystal structure (6M17). (**B** & **C**) Molecular docking of human ACE2 and EGCG. (**B**) Three-dimensional representation of EGCG binding with the human ACE2 peptidase domain (left) and magnified image (right). (**C**) Two-dimensional interaction analysis of ACE2 and EGCG, highlighting the ACE2 residues that interact with EGCG and the types and lengths of their bonds. Residues are color coded according to the type of interaction: dark green, conventional hydrogen bonds; light green, van der Waals forces; dark pink, Pi–Pi stacked interactions; and light pink, Pi–alkyl interactions.

We investigated the potential effect of EGCG to interact with ACE2 and block its binding to the spike RBD. In this context, previous structural analyses of the RBD of the SARS-CoV-2 spike and peptidase domain of ACE2 provided a platform aiming to assess the different interactions of their interface residues within a 5-Å region [10, 25, 26]. For this reason, we identified the key residues that could serve as crucial interaction sites of SARS-CoV-2 spike RBD and ACE2 (Fig 3A). Next, to determine its ability to target ACE2, we performed *in silico* docking of EGCG. The three-dimensional and two-dimensional interaction diagrams of EGCG with ACE2 indicated the binding pattern. Therefore, we identified the key interacting residues (Fig 3B and 3C); the binding score was −7.8 kcal/mol, and the length of the hydrogen bond with R393 was 2.95 Å (Fig 3C). The *in silico* results indicated that EGCG binding with ACE2 might block the interaction between the SARS-CoV-2 spike RBD and ACE2.

### Caco-2 cells express ACE2 and 2-h treatment of EGCG did not significantly suppress Caco-2 cell viability

**Fig 4:**
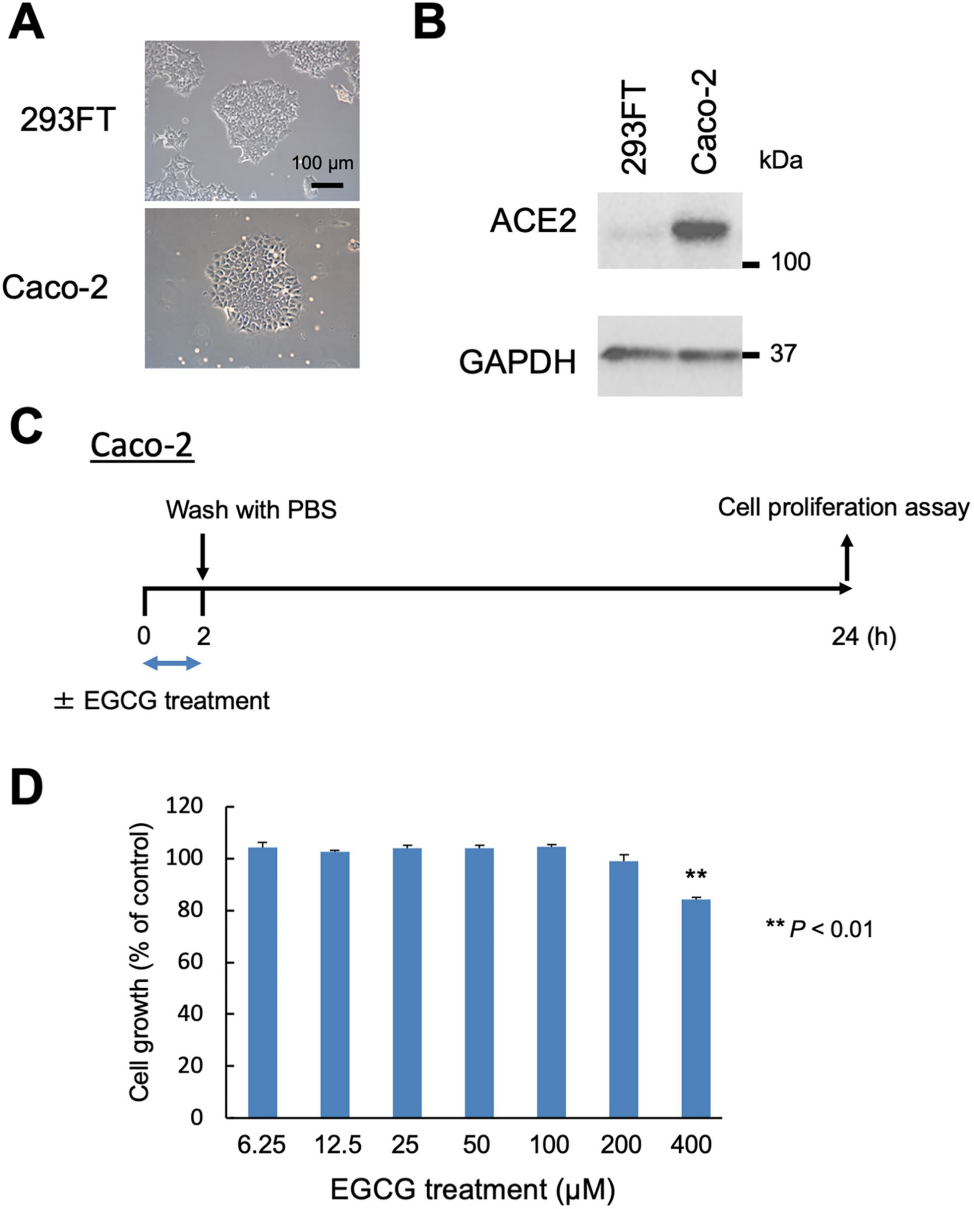
Effects of epigallocatechin gallate (EGCG) treatment for 2 h on the growth of angiotensin-converting enzyme 2 (ACE2)-receptor expressing Caco-2 cells. (**A**) Images of 293FT and Caco-2 cells grown on 100-mm plates taken under a phase-contrast microscope. Scale bar: 100 μm. (**B**) Cropped image of western blot performed with ACE2 and Glyceraldehyde 3-phosphate dehydrogenase antibodies. (**C**) Outline of the experimental procedure. Caco-2 cells were treated with or without EGCG for 2 h. After treatment, the cells were washed with PBS thrice, and fresh medium was added. After 22 h of incubation, cell growth was determined. (**D**) Effects of EGCG on Caco-2 cell growth. Cell growth was assessed by the cell proliferation assay and expressed as a percentage of dimethyl sulfoxide (DMSO)-treated control cells. Asterisks indicate the significant difference compared with the DMSO-treated control (**p < 0.01).

Recently, the human colorectal adenocarcinoma cell line, Caco-2, was reported to be infected by SARS-CoV-2 *in vitro* using the ACE2 receptor for entry; nevertheless, the infection was negative in the human embryonic kidney cell line, 293FT [32–34]. Here we examined the expression of ACE2 in Caco-2 cells via western blotting. As expected, ACE2 expression was confirmed in Caco-2 cells but was absent in 293FT cells (Fig 4A and 4B). To examine the effects of EGCG on the growth of Caco-2 cells *in vitro*, we performed the CellTiter-Glo assay to analyze the cell viability. Caco-2 cells were treated with various concentrations (6.25, 12.5, 25, 50, 100, 200, and 400 μM) of EGCG for 2 h. As a result, we can confirm that the concentrations below 200 μM of EGCG exhibited no significant effect on cell viability, whereas the cell growth was reduced significantly at the concentration of 400 μM of EGCG (*p* < 0.01) (Fig 4C and 4D). Therefore, a treatment with EGCG ≤ 200 μM for 2 h did not significantly suppress Caco-2 cell viability.

### EGCG inhibits SARS-CoV-2 infection in Caco-2 cells

**Fig 5:**
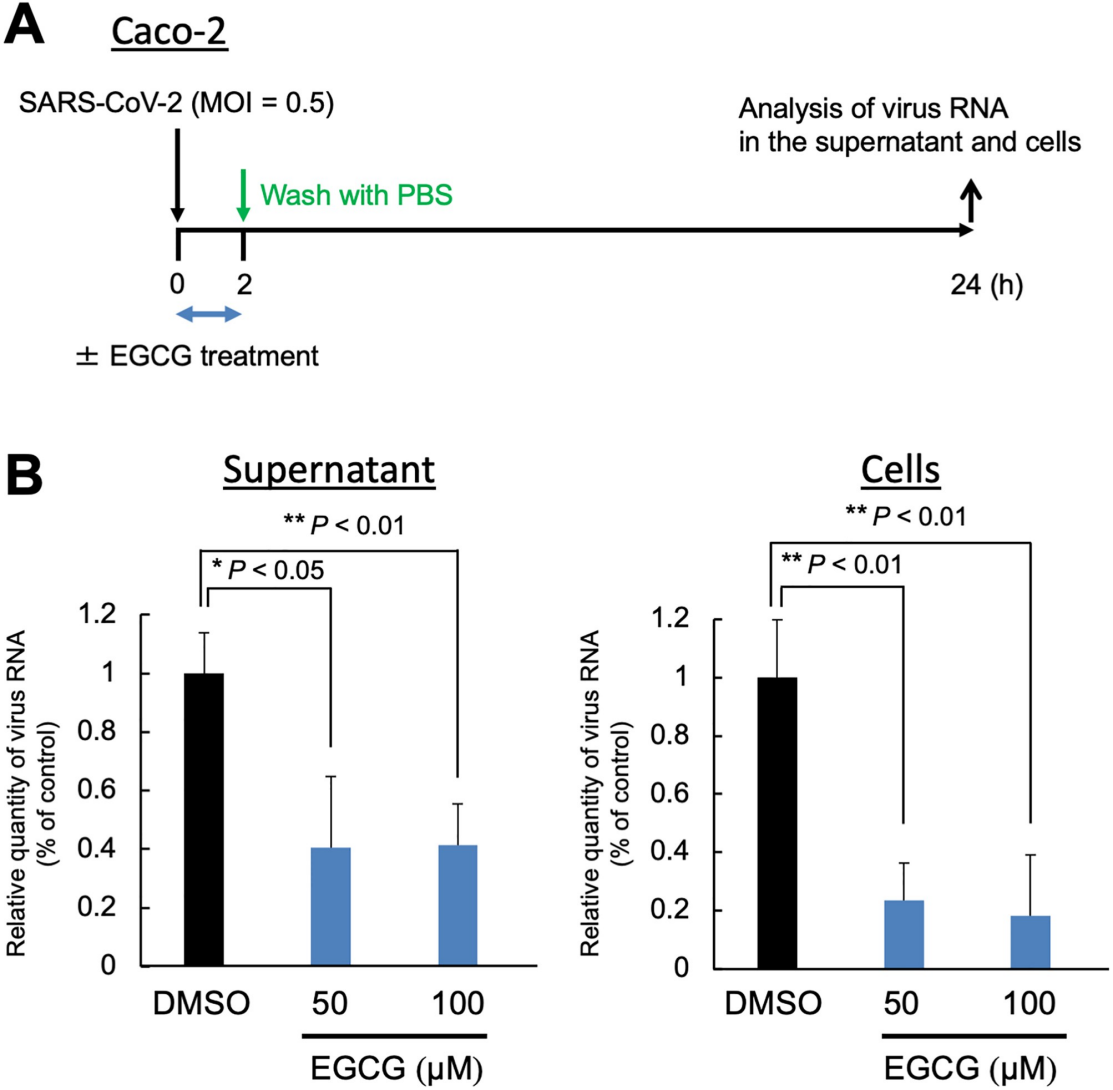
Epigallocatechin gallate (EGCG) inhibits severe acute respiratory syndrome coronavirus 2 (SARS-CoV-2) infection and replication in Caco-2 cells. (**A**) Schematic outline of the experimental procedure. SARS-CoV-2 (MOI 0.5)-infected Caco-2 cells were co-cultured with EGCG at a final concentration of 50 or 100 μM. At 2 hpi, the cells were washed with phosphate-buffered saline thrice, and fresh medium was added. At 24 hpi, the cell culture supernatant and cell lysate were collected, and the amount of viral RNA was analyzed by real-time reverse transcription-polymerase chain reaction (RT-qPCR). (**B**) Measurement of the SARS-CoV-2 growth inhibition by EGCG determined by RT-qPCR of the nucleocapsid region of the SARS-CoV-2 genome using the supernatant (left) or cells (right). Values are presented as a percentage of control (mean ± SD). Asterisks indicate the significant difference compared with the dimethyl sulfoxide–treated control (**p < 0.01, *p < 0.05).

Based on the ELISA results and the *in silico* docking study, we identified EGCG as a potent inhibitor of SARS-CoV-2 spike RBD binding to ACE2 (Figs 2B and 3). Next, we investigated whether EGCG could inhibit SARS-CoV-2 infection *in vitro*; we used Caco-2 cells as target cells of SARS-CoV-2 infection due to the endogenous expression of ACE2 (Fig 4A and 4B). We co-cultured the Caco-2 cells infected by SARS-CoV-2 with EGCG at 50 μM and 100 μM for 2 h. Then, we measured the intracellular viral RNA levels after incubation for 22 hpi (Fig 5A). The results showed that EGCG treatment significantly reduced the synthesis of SARS-CoV-2 RNA, achieving 59.4%–58.6% inhibition in the supernatant and 76.4%–81.8% inhibition in the cells at 50 μM–100 μM EGCG, respectively (Fig 5B). To investigate the influence of EGCG at the fusion/entry stages of viral replication, we measured the intracellular viral RNA levels at 2 hpi. As shown in S2 Fig, the 100-μM EGCG treatment significantly reduced the SARS-CoV-2 RNA (*p* < 0.05), achieving 44% inhibition of the cells.

### EGCG pretreatment attenuates SARS-CoV-2 infection in Caco-2 cells

A recent *in vitro* study showed that EGCG inhibits SARS-CoV-2 3-chymotrypsin-like protease (3CLpro), which is a virus protease essential for proteolytic function in the maturation stage of the virus, also known as main protease (Mpro) or nonstructural protein 5 (Nsp5) [35–38]. To further examine whether the antiviral effect of EGCG is direct or indirect effect on SARS-CoV-2 itself, we measured the intracellular viral RNA levels after 2 h of pretreatment (indirect effect) and cotreatment (direct effect) with EGCG at 100 μM (S3A Fig). As a result, both treatments with EGCG significantly reduced the synthesis of SARS-CoV-2 RNA, achieving inhibition of 55.3% and 37.0% in the supernatant and 75.2% and 29.6% in the cells, in pretreatment and cotreatment, respectively (S3B Fig). Consequently, these results suggested that EGCG could attenuate SARS-CoV-2 infection through not only direct effects but also indirect effects on SARS-CoV-2.

## Discussion

COVID-19 developed into a global pandemic and markedly increased the rates of morbidity and mortality in many countries [39]. Therefore, developing therapeutic and/or preventive drugs against COVID-19 is crucial to suppress this disease quickly and effectively. In this study, we selected many common phytochemicals found in various plants, based on evidences showing their potential bioactivities for treating several diseases, such as rheumatoid arthritis and cancer [40, 41]. The aim of our study was to identify an appropriate natural compound that could target the interaction of the SARS-CoV-2 spike protein and the cellular receptor ACE2 in human cells. In this context, we applied ELISA-based screening to investigate the effects of 10 phytochemicals on this key interaction in SARS-CoV-2 pathogenicity and identified EGCG as the one that exerted the most significant inhibitory effect.

Similar to SARS-CoV, the interaction between the RBD of SARS-CoV-2 spike with the peptidase domain of human ACE2 in host cells has been recently studied in detail at the structural level [10, 25, 33, 42]. However, few natural drug molecules were found to effectively bind and disrupt this interaction [43–47]. Interestingly, the docking study results of target protein–ligands and their interaction analyses revealed that EGCG binding might inhibit the SARS-CoV-2 spike RBD interaction with ACE2 (Fig 3). These findings suggest that EGCG might be a potential candidate in developing an initial structure that acts as an inhibitor of SARS-CoV-2 infection.

EGCG is easily extracted from the leaves of the plant *Camellia sinensis*, which is consumed worldwide [48]. In addition, EGCG is the most abundant and pharmacologically potent catechin found in green tea [49]; a cup of green tea brewed from 2.5 g of tea leaves contain 240–320 mg of catechin polyphenols, out of which EGCG accounts for 60%–65% [50]. Nevertheless, EGCG is thought to be mostly responsible for a broad of beneficial activities of green tea on human health, such as fighting against cancer, obesity, diabetes, cardiovascular, infectious, and neurodegenerative diseases [14, 16, 17, 51–53]. From another side, cumulative evidences indicated that the binding affinity of EGCG to specific proteins is involved in its mechanism of action [50, 54–56]. Therefore, EGCG, or any version chemically modified appropriately with an improved efficiency, might interfere with the interaction between SARS-CoV-2 spike protein and ACE2.

Currently, various approaches have been suggested to inhibit SARS-CoV-2 infection, including the use of neutralizing antibodies, vaccines, and small molecules [7, 57], among which, vaccines are considered to be the most effective, although they may not be equally feasible in all countries given that their development and production are costly and time consuming. For this reason, finding potent, easy to prepare, and cheap agents that could effectively inhibit SARS-CoV-2 infection at early stages is preferable. Because SARS-CoV-2 spike protein binds to ACE2 via RBD at an early stage, disrupting the interaction between the RBD and ACE2 is considered the main target in order to prevent SARS-CoV-2 infection and viral replication [58–60]. Here, we used ELISA-based screening and identified two phytochemicals (curcumin and EGCG) that can block this interaction. Nevertheless, EGCG exhibited greater inhibitory activity than curcumin; therefore, we focused on the use of EGCG in the SARS-CoV-2 infection experiment. Consequently, EGCG significantly attenuated the entry of SARS-CoV-2 into target cells *in vitro* and/or blocked viral replication.

Accumulating evidences suggested that EGCG inhibits SARS-CoV-2 3CLpro [35–38]. Moreover, a molecular docking study showed that EGCG bound several SARS-CoV-2 target proteins including 3CLpro and spike RBD [61]. Furthermore, Henss et al. revealed that EGCG inhibited SARS-CoV-2 virus replication and interfered with the interaction between the SARS-CoV-2 spike protein and ACE2 [62]. In this study, we showed that not only EGCG co-treatment but also pretreatment significantly reduced the infection of SARS-CoV-2 into Caco-2 cells (S3B Fig). Although the strong inhibitory effect of EGCG co-treatment on SARS-CoV-2 infection might be due to the direct action of EGCG on SARS-CoV-2 itself [35–38], EGCG pretreatment also significantly reduced the infection (S3B Fig). Despite we could not identify the molecular target of EGCG that inhibits SARS-CoV-2 entry into Caco-2 cells, the findings of this study might help to further elucidate the mechanisms of the anti-SARS-CoV-2 effects of EGCG. This study might be the first step toward developing inhibitory agents to block SARS-CoV-2 entry into host cells.

## Supporting information

S1 Fig Epigallocatechin gallate (EGCG) (20 μM) inhibits the interaction between the spike receptor-binding domain (RBD) and human cell receptor angiotensin-converting enzyme 2 (ACE2). Enzyme-linked immunosorbent assay (ELISA) results of phytochemical inhibition of the ACE2 and spike RBD binding. Low-intensity horseradish peroxidase signals indicate that the compound successfully blocked the ACE2 and spike RBD binding. Values are presented as mean ± SD. Asterisks indicate a significant difference compared with the dimethyl sulfoxide–treated control (**p < 0.01).
(TIFF)

S2 Fig Epigallocatechin gallate (EGCG) inhibits severe acute respiratory syndrome coronavirus 2 (SARS-CoV-2) infection in Caco-2 cells. (**A**) Schematic outline of the experimental procedure. SARS-CoV-2 (MOI 1)-infected Caco-2 cells were cocultured with EGCG at a final concentration of 100 μM. At 2 hpi, the cell lysates were collected, and the amount of viral RNA was analyzed by real-time reverse transcription-polymerase chain reaction (RT-qPCR). (**B**) Measurement of the infected SARS-CoV-2 inhibition by EGCG determined by RT-qPCR of the nucleocapsid region of the SARS-CoV-2 genome. Values are presented as percentage of control (mean ± SD). Asterisks indicate a significant difference compared with the dimethyl sulfoxide–treated control (*p < 0.05).
(TIFF)

S3 Fig Pretreatment and cotreatment of epigallocatechin gallate (EGCG) attenuate severe acute respiratory syndrome coronavirus 2 (SARS-CoV-2) infection and replication in Caco-2 cells. (**A**) Schematic outline of the experimental procedure. SARS-CoV-2 (MOI 0.5)-infected Caco-2 cells were pre-treated or co-treated with EGCG at a concentration of 100 μM. Before or after 2 h SARS-CoV-2 infection, the cells were washed with phosphate-buffered saline thrice, and fresh medium was added. At 24 hpi, the cell culture supernatant and cell lysate were collected, and the amount of viral RNA was analyzed by real-time reverse transcription-polymerase chain reaction (RT-qPCR). (**B**) Measurement of the SARS-CoV-2 growth inhibition by EGCG determined by RT-qPCR of the nucleocapsid region of the SARS-CoV-2 genome using the supernatant (left) or cells (right). Values are presented as a percentage of control (mean ± SD). Asterisks indicate the significant difference compared with the dimethyl sulfoxide–treated control (**p < 0.01).
(TIFF)

S1 Raw images (TIFF)
