# Supplementary figures and images for: Epigallocatechin gallate (EGCG) attenuates severe acute respiratory coronavirus disease 2 (SARS-CoV-2) infection by blocking the interaction of SARS-CoV-2 spike protein receptor-binding domain to human angiotensin-converting enzyme 2
Source: PLoS One. 2022 Jul 13;17(7):e0271112. doi: 10.1371/journal.pone.0271112 (PMC9278780; doi:10.1371/journal.pone.0271112)

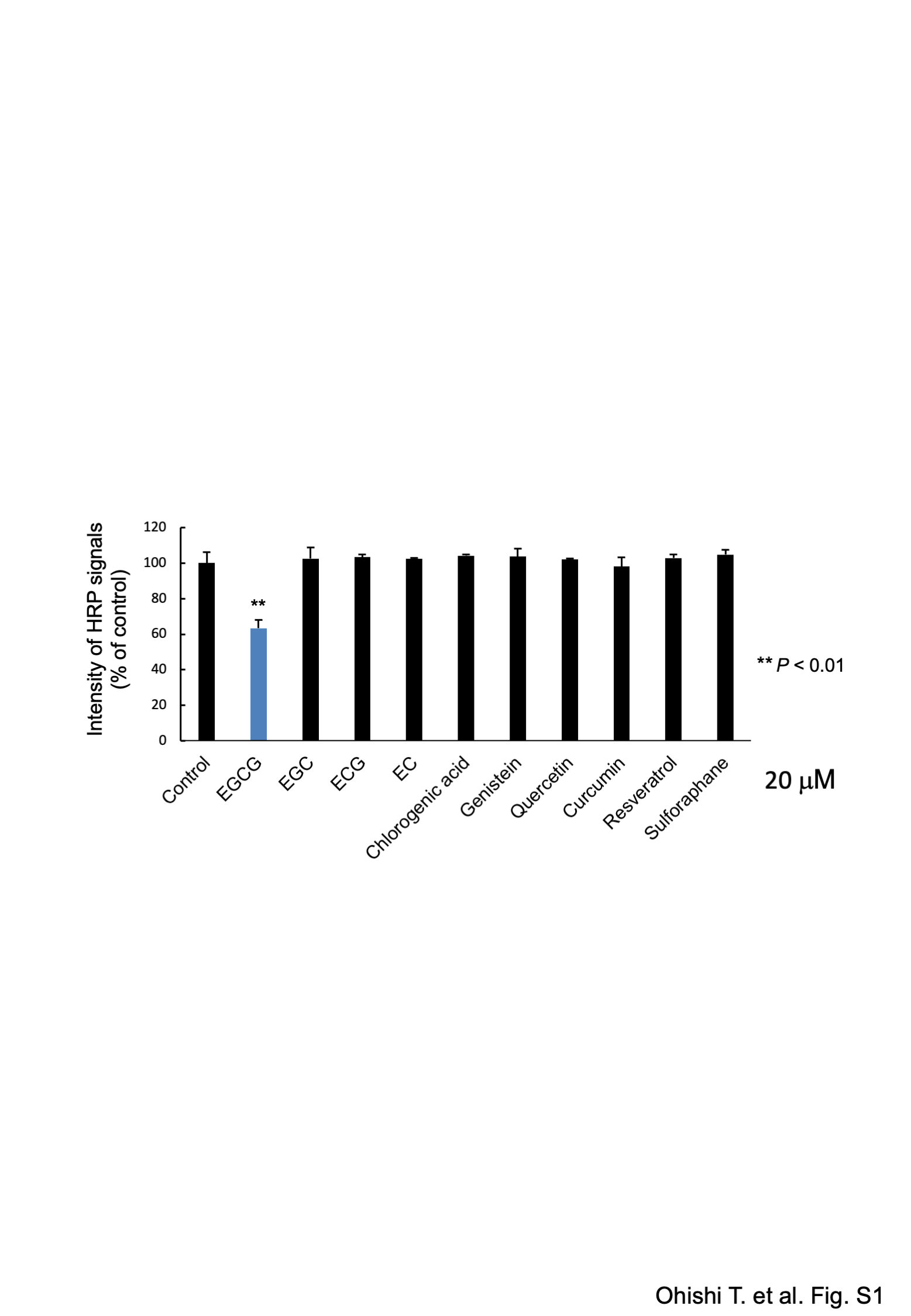

Supplement: S1 Fig — Enzyme-linked immunosorbent assay (ELISA) results of phytochemical inhibition of the ACE2 and spike RBD binding. Low-intensity horseradish peroxidase signals indicate that the compound successfully blocked the ACE2 and spike RBD binding. Values are presented as mean ± SD. Asterisks indicate a significant difference compared with the dimethyl sulfoxide–treated control (**p < 0.01). (TIFF) [file pone.0271112.s001.tiff]

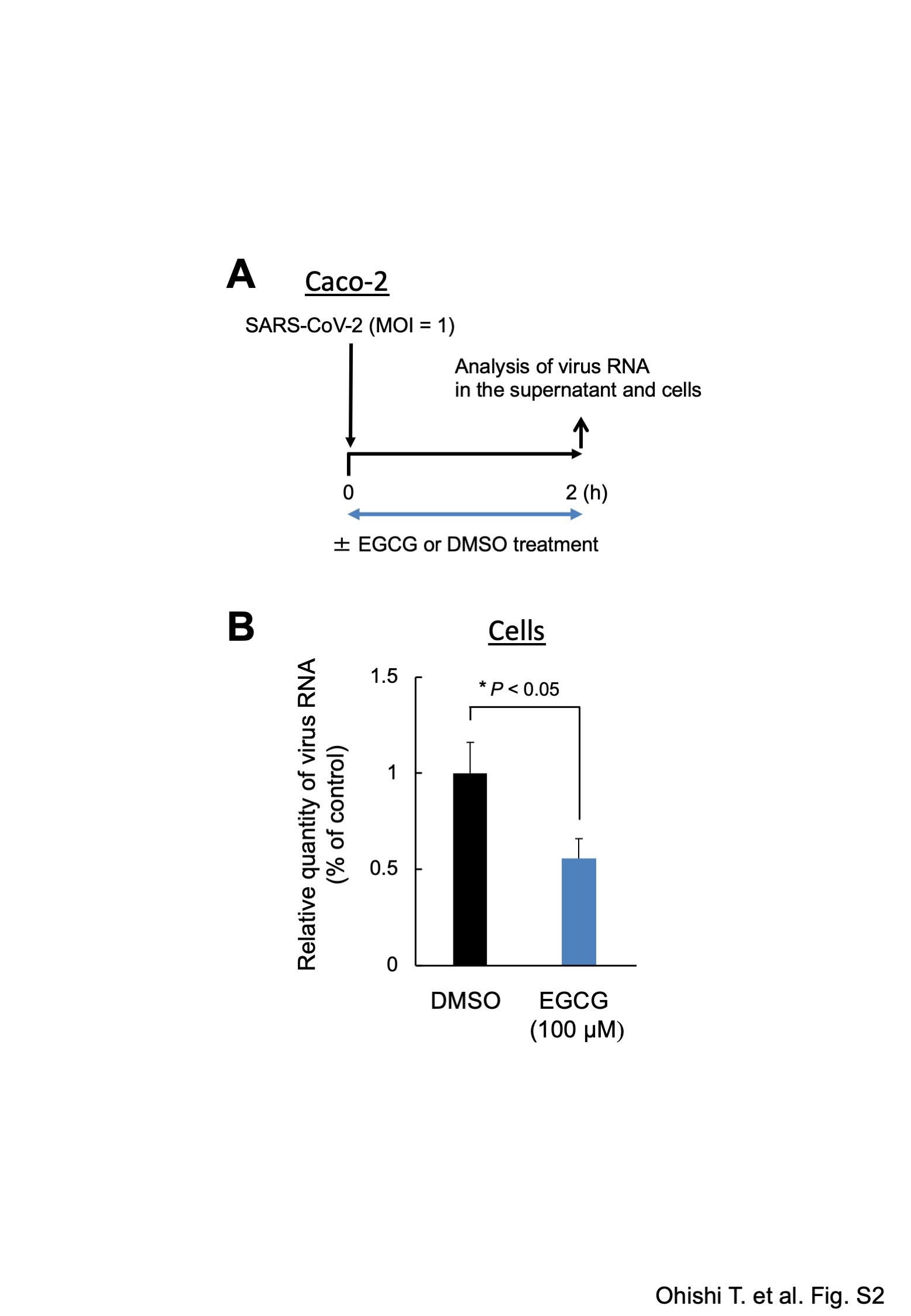

Supplement: S2 Fig — (A) Schematic outline of the experimental procedure. SARS-CoV-2 (MOI 1)-infected Caco-2 cells were cocultured with EGCG at a final concentration of 100 μM. At 2 hpi, the cell lysates were collected, and the amount of viral RNA was analyzed by real-time reverse transcription-polymerase chain reaction (RT-qPCR). (B) Measurement of the infected SARS-CoV-2 inhibition by EGCG determined by RT-qPCR of the nucleocapsid region of the SARS-CoV-2 genome. Values are presented as percentage of control (mean ± SD). Asterisks indicate a significant difference compared with the dimethyl sulfoxide–treated control (*p < 0.05). (TIFF) [file pone.0271112.s002.tiff]

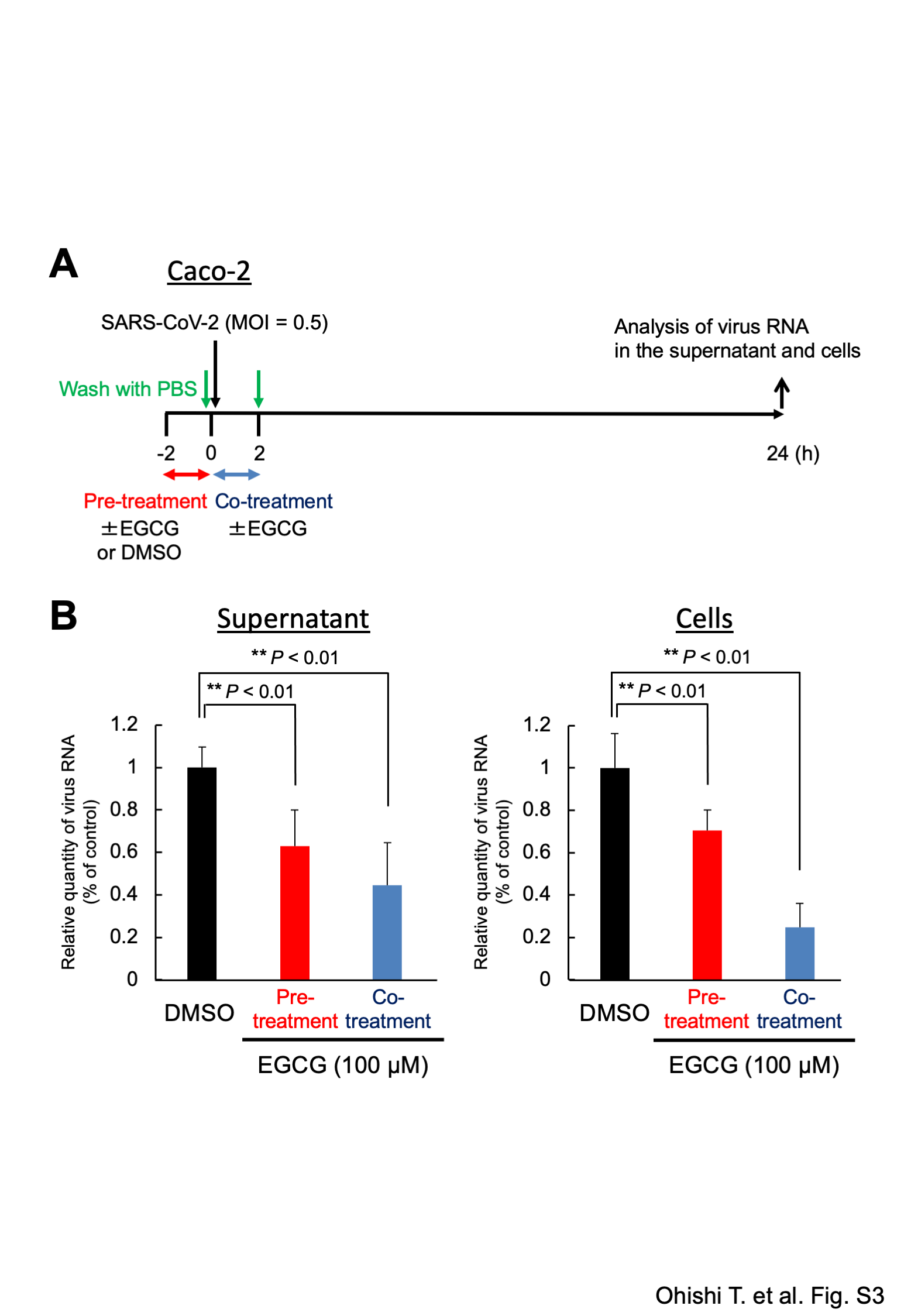

Supplement: S3 Fig — (A) Schematic outline of the experimental procedure. SARS-CoV-2 (MOI 0.5)-infected Caco-2 cells were pre-treated or co-treated with EGCG at a concentration of 100 μM. Before or after 2 h SARS-CoV-2 infection, the cells were washed with phosphate-buffered saline thrice, and fresh medium was added. At 24 hpi, the cell culture supernatant and cell lysate were collected, and the amount of viral RNA was analyzed by real-time reverse transcription-polymerase chain reaction (RT-qPCR). (B) Measurement of the SARS-CoV-2 growth inhibition by EGCG determined by RT-qPCR of the nucleocapsid region of the SARS-CoV-2 genome using the supernatant (left) or cells (right). Values are presented as a percentage of control (mean ± SD). Asterisks indicate the significant difference compared with the dimethyl sulfoxide–treated control (**p < 0.01). (TIFF) [file pone.0271112.s003.tiff]

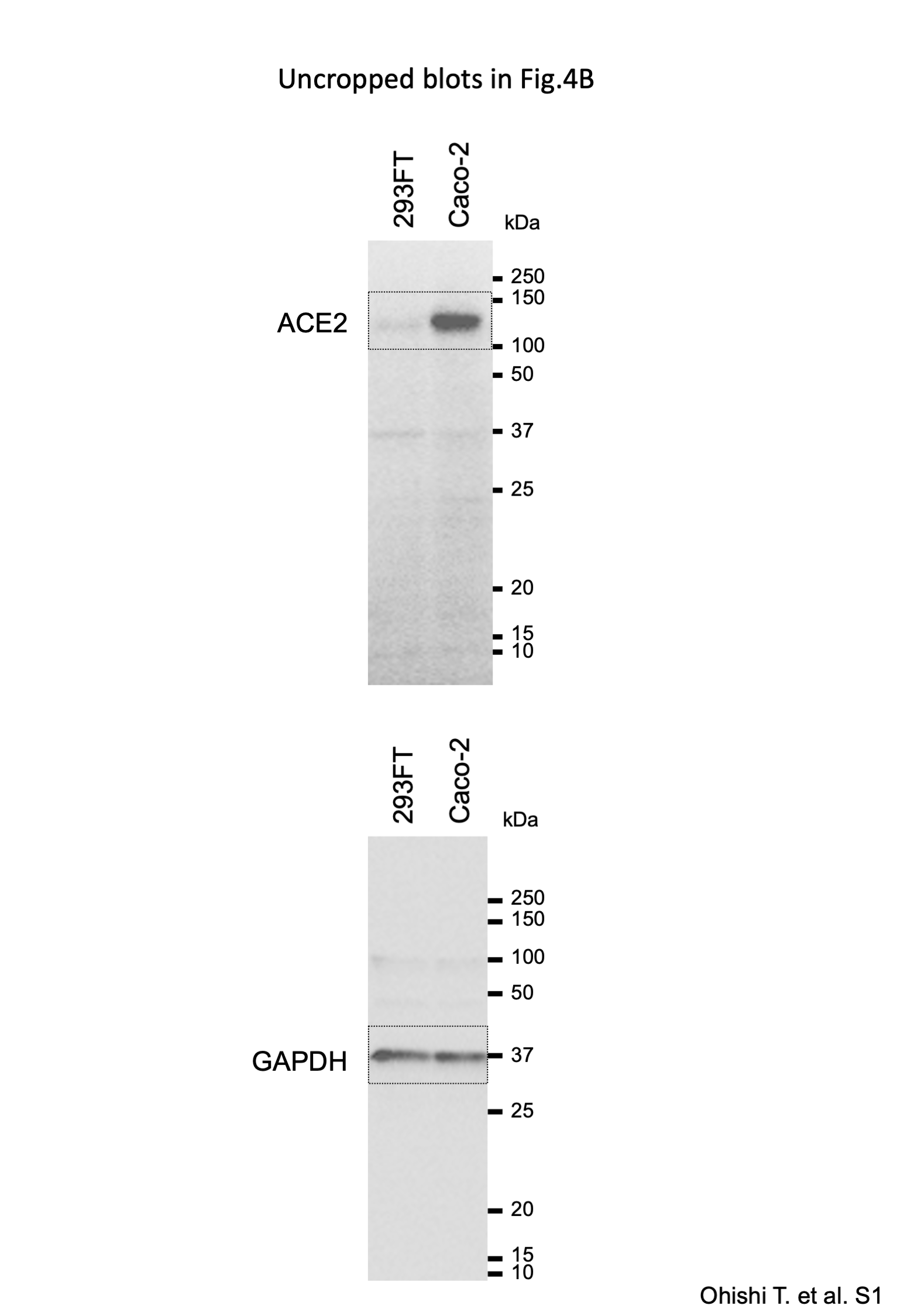

Supplement: S1 Raw images — (TIFF) [file pone.0271112.s004.tiff]
